# Metalens-Based Miniaturized Optical Systems

**DOI:** 10.3390/mi10050310

**Published:** 2019-05-08

**Authors:** Bo Li, Wibool Piyawattanametha, Zhen Qiu

**Affiliations:** 1 Department of Electrical and Computer Engineering, Michigan State University, East Lansing, MI 48823, USA; libo2@msu.edu; 2 Institute for Quantitative Health Science and Engineering, Michigan State University, East Lansing, MI 48823, USA; wibool@gmail.com; 3 Department of Biomedical Engineering, Faculty of Engineering King Mongkut’s Institute of Technology Ladkrabang (KMITL), Bangkok 10520, Thailand; 4 Department of Biomedical Engineering, Michigan State University, East Lansing, MI 48823, USA

**Keywords:** metasurface, metalens, field of view (FOV), achromatic, Huygens’ metalens, bio-optical imaging, optical coherence tomography, confocal, two-photon, spectrometer

## Abstract

Metasurfaces have been studied and widely applied to optical systems. A metasurface-based flat lens (metalens) holds promise in wave-front engineering for multiple applications. The metalens has become a breakthrough technology for miniaturized optical system development, due to its outstanding characteristics, such as ultrathinness and cost-effectiveness. Compared to conventional macro- or meso-scale optics manufacturing methods, the micro-machining process for metalenses is relatively straightforward and more suitable for mass production. Due to their remarkable abilities and superior optical performance, metalenses in refractive or diffractive mode could potentially replace traditional optics. In this review, we give a brief overview of the most recent studies on metalenses and their applications with a specific focus on miniaturized optical imaging and sensing systems. We discuss approaches for overcoming technical challenges in the bio-optics field, including a large field of view (FOV), chromatic aberration, and high-resolution imaging.

## 1. Introduction

Miniaturized optical systems, for both imaging and sensing, have recently become very attractive for many biomedical applications, such as wearable and endoscopic medical devices. Novel optical lenses with ultrathin structure and light weight have played an important role in the miniaturization of state-of-the-art bio-optical systems. Traditional planar optical lenses (such as micro-gratings and Fresnel micro-lenses) and thin-film micro-optics have been studied in the last few decades. Although the device’s footprint has been slightly reduced by using these lenses, conventional lenses have already been shown to have many disadvantages, including limited optical quality for imaging, integration difficulties, and high cost. Metasurface-based flat optical lenses (so-called metalenses) [1,2,3,4,5,6,7] show great potential and could overcome most of the challenges. The meta building blocks (MBBs) work as subwavelength-spaced scatterers. Many basic properties of light [8,9,10] (such as phase, polarization, and focal points) can be controlled in high-resolution imaging and sensing, through tuning the MBBs’ shapes, size, and positions. Conventional lenses, such as refractive lenses (objectives and telescope), are usually bulky and expensive, although they are still dominant in optical systems. Unfortunately, their fabrication processes (such as molding, polishing, and diamond-turning) are commonly sophisticated. In addition, the phase profiles are quite limited, while the structure of the lenses is small. On the contrary, metalenses overcome those limitations and provide great advantages compared to traditional optical elements. Especially by using accurate numerical methods, the phase profiles of metalenses can be well designed with MBBs. With advanced micro-machining processes, metalenses can be mass-produced with high yield.

## 2. The Fundamentals of Metasurface-Based Lenses

### 2.1. Phase Profile Control

Refractive lenses are widely used in various optical systems such as telescopes and microscopes. Although they have very good properties in phase control and polarization, traditional refractive lenses with a high numerical aperture (NA) are often bulky and expensive. Additionally, the complex macro- or meso-scale fabrication process still relies on conventional optics manufacturing methods, which have been developed for over 100 years. To meet the optical requirements, refractive lenses are usually designed with different shapes. However, a metalens provides new opportunities to overcome these limitations. For instance, the phase profile can be modified by changing the MBBs [11]. The hyperbolic phase profile [2] required for focusing a normal incident beam that remains collimated inside the substrate can be expressed as follows:

(1) $$\varphi \left(r\right)\;=\;-\frac{2\pi }{\lambda }\left(\sqrt{r^{2}+f^{2}}-f\right)$$

where *f* is the focal length of the illumination wavelength and *r* is the radial coordinate. The designed metasurface should create a phase profile to modulate the incident planar wavefront into spherical ones at focal length *f* from the lenses.

### 2.2. Plasmonic Metasurface-Based Lenses

Usually, metasurface-based lenses use MBBs to modify the optical characteristics. One of the most representative techniques is to create plasmonic effects on the surface. A plasmonic antenna [12] can be easily micro-machined using advanced electron beam lithography (EBL) and a relatively simple lift-off process. The concentrated incident light can be transformed into a smaller region that matches its own wavelength and causes oscillations. By having the plasmonic effect on its metasurface, the metalens has attracted great interest in the optics field. For example, experimental results reported by Yin et al. [13] have indicated that the micro-structures have a plasmonic effect on the Ag film surface and successfully formed a focal spot at the focusing plane. Another study, by Zhang et al. [14], has shown that the focusing could be achieved and also tuned using different nano-antenna shapes such as elliptical and circular blocks.

### 2.3. All Dielectric Metasurface-Based Lenses

While dielectric phase shifters are utilized in the MBBs, the energy absorption loss of the incident could potentially be reduced significantly. Researchers have taken advantage of this and included dielectric phase shifters in many new optics designs. For example, Vo et al. [10] proposed polarization independent lenses with dielectric building blocks (circular silicon arrays). High transmission efficiency (70%) has been achieved with the incident light at a wavelength of 850 nm. Faraon’s group from Caltech [15] has demonstrated that a single dielectric nano-antenna could be designed as an efficient building block that might provide full phase coverage. Based on the optimized nanostructures, the spatial image resolution has excellent qualities and relatively high transmission efficiency. Capasso’s group from Harvard University [16] has demonstrated that the dielectric metalens also has superior performance in spectral applications in the visible range. The polarization independent metalens has been micro-fabricated by titanium dioxide (TiO_2_) nanopillars. The metalens could achieve a relatively high NA = 0.85 with an efficiency of more than 60% for incident wavelengths of 532 nm and 660 nm.

## 3. Advanced Techniques for Metasurface-Based Lenses

### 3.1. Wide Angular Field of View

In a miniaturized optical system, the field of view (FOV) is one of the key factors for evaluating the overall qualities of the imaging and sensing system [17]. Unfortunately, due to technical limitations, most metalenses suffer from serious off-axis aberration, leading to a limited FOV. Pursuing increased FOV is a common goal of many scientific studies. For example, in traditional optical lens-based imaging systems, bulky and expensive aberration-corrected objective lenses are frequently utilized to achieve a relatively large FOV.

**Figure 1:**
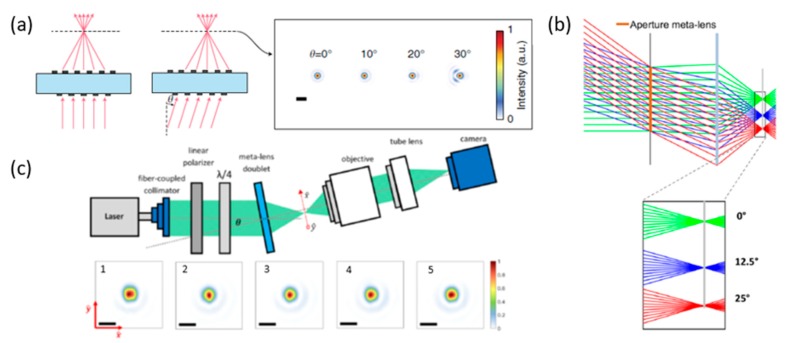
Focal spot characterization for different angles of incidence source. (**a**) (**left**) Schematic illustration of focusing of on-axis and off-axis light by a metasurface doublet lens. (**right**) Simulated focal plane intensity for different incident angles. (Reproduced from [24] with permission.) (**b**) The Ray diagram obtained by adding the aperture meta-lens resulting in diffraction-limited focusing along the focal plane. (**c**) Focal spot measurement setup. (1–5) Focal spot intensity profile at (1) 0°, (2) 6°, (3) 12°, (4) 18°, (5) 25° incidence angle. (Reproduced from [25] with permission.)

Theoretically, single-layer metasurface-based flat lenses suffer from off-axis aberrations [18,19], along with wide-angle absorption [20,21,22,23] and other problems. To broaden the FOV, multiple-layer metalens structures have been successfully demonstrated. For example, Faraon’s group [24] has shown a doublet lens formed by cascading two metasurfaces (Figure 1a), which could achieve limited diffraction, focusing up to ± 30° with near-infrared (NIR) incident light of 850 nm. For a shorter wavelength (532 nm) in the visible range, Capasso’s group [25] reported a metalens doublet design. For the aperture metalens, shown in Figure 1b, with positive and negative angle incident light, the spherical aberration can be corrected and all the focusing points can eventually be allocated on the same focal plane. Based on the principle of the Chevalier lens [26], the metalens shown in Figure 1c has provided a relatively larger FOV with an incident light angle up to ±25°.

**Figure 2:**
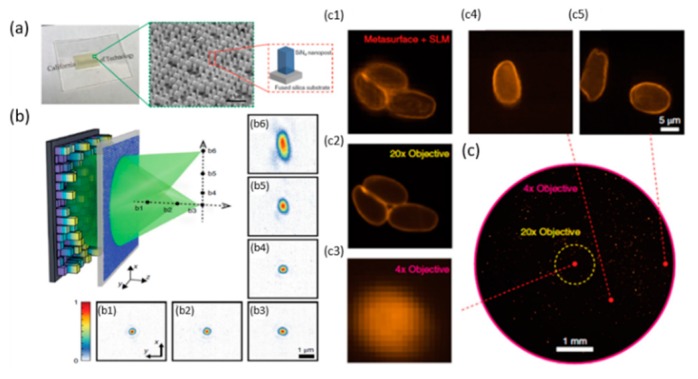
Disorder-engineered metasurfaces. (**a**) Photograph and SEM image of a fabricated disorder-engineered metasurface. (**b**) Schematic of optical focusing assisted by the disordered metasurface. The incident light is polarized along the *x* direction (b1–b6). (**c**) Low-resolution bright-field image captured by a conventional fluorescence microscope with a 4 × objective lens (NA = 0.1). (Reproduced from [27] with permission.)

Most recently, single-layer metalens with disorder-engineered design [27] providing the optical randomness of conventional disordered media, but in a way that is fully known a priori (Figure 2a), has been demonstrated by Yang and Faraon’s groups at Caltech [27] with improved resolution and FOV. This disorder-engineered metalens has individual input‒output responses, which is different from the multiple-lens based system. The new metalens has a high numerical aperture (NA ~0.5) focusing to 2.2 × 10^8^ points in an ultra-large FOV with an outer diameter of 8 mm.

**Figure 3:**
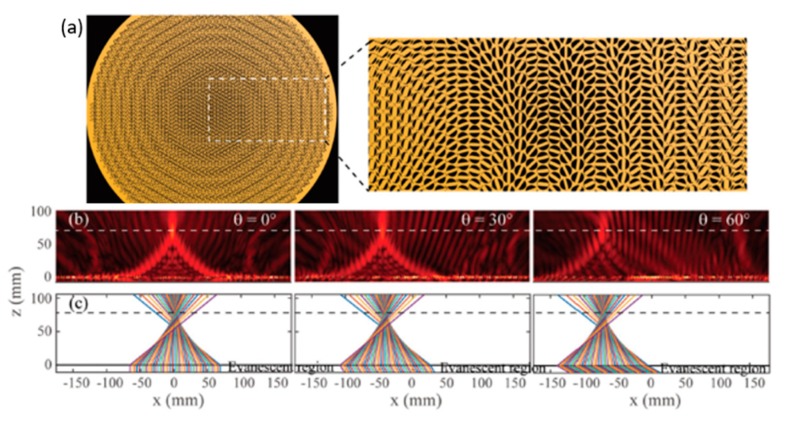
Performance of the wide-angle flat lens. (**a**) Perspective and zoom view of the wide-angle flat lens. (**b**) Simulated light intensity distributed on the xoz plane at 19 GHz electric field distributions. (**c**) Ray trajectories of 19 GHz before and after propagating through the flat lens. Left, middle, and right panels of (**b**) and (**c**), respectively, represent the cases of ∅ = $0^{{}^{\circ}}$, $30^{{}^{\circ}}$, $60^{{}^{\circ}}.$ (Reproduced from [28] with permission.)

To increase the FOV, another approach has been proposed by Guo et al. [28]. The key to constructing a metalens with larger FOV is to realize a perfect conversion from rotational symmetry to translational symmetry in the light field [28]. The wavenumber in free space and incident angles should satisfy the following relation, shown as Equation (2):

(2) $$k_{0}sin\theta_{x}x+k_{0}sin\theta_{y}y+\emptyset_{m}\left(x,y\right)\;=\;\emptyset_{m}\left(x+\Delta_{x},\;y+\Delta_{y}\right)$$

where the $k_{0}$ is the wavenumber in free space, $\emptyset_{m}\left(x,y\right)$ is the phase shift profile carried by the flat lens, $\Delta_{x}$ and $\Delta_{y}$ correspond to the translational shift of $\emptyset_{m}\left(x,y\right)$ at incidence angles of $\theta_{x}$ and $\theta_{y}$ [28]. To verify this method, a new metalens with diameter of 350 mm and focal length *f* = 87.5 mm (NA ~ 0.89) was simulated at 19 GHz, shown in Figure 3a. As a proof of concept, the measurement of far-field power patterns is demonstrated with a circularly polarized horn through the metalens for wide FOV. The result shows that a ± 60° beam steering can be realized by the transversely changing the location of the antenna within an area from − 75.8 mm to + 75.8 mm.

**Figure 4:**
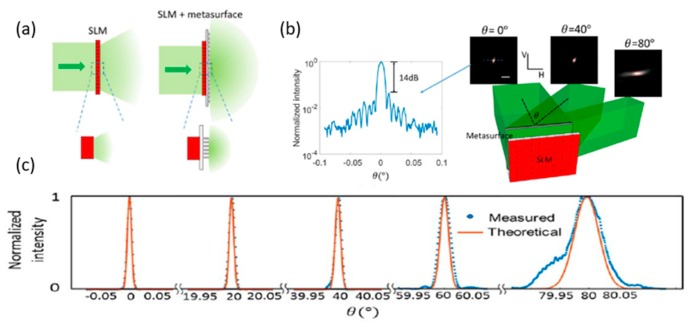
Wide-angular-range and high-resolution beam steering by a metasurface-coupled phased array. (**a**) The comparison of steering range of a single SLM structure and a metasurface-coupled SLM structure. (**left**) without the metasurface, the SLM can provide only a small diffraction envelope. (**right**) With the metasurface-coupled SLM structure, since each scatterer is subwavelength, the steerable range can span from −90° to +90°. (**b**) Illustration of the steering scheme (**c**) 1D far−field beam shapes at other steering angles. Red lines denote the theoretical shapes of the beams. Blue dots denote the measured data. (Reproduced from [29] with permission.)

To achieve broader FOV and preserve high-resolution imaging performance, Yang and Faraon’s groups [29] have demonstrated a new phase-array-based method (Figure 4) that does not require a large scale-up in the number of controllable elements [29]. It uses disorder-engineered design, as the key factor is similar to that of previous work [27].

The 2D array subwavelength scatters (SiN_x_ with height 630 nm) were deposited on the silica substrate arranged in a square lattice with a pitch size of 350 nm. Also, when the designed lens combined with a spatial light modulator (SLM), the output light of the system would have a larger cover angle range than what is possible with a SLM alone. As a result, the disorder-engineered metasurface with SLM was able to scatter light uniformly within the range of ± 90° (Figure 4a) due to the subwavelength size and random distribution of the nanofins.

Based on the above introduction, the FOV of optical systems can be increased by using proper metasurface design. The nano-element size, material and metasurface structure have different effects to the selected incident light in improving the FOV. A comparison table of different metasurface designs for improving the FOV is shown below (Table 1).

### 3.2. Achromatic Metasurface-Based Lenses

**Figure 5:**
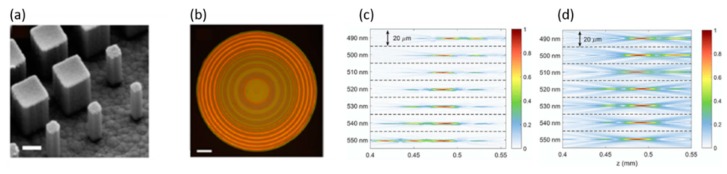
A 60 nm bandwidth achromatic metalens. (**a**) Side view scanning electron microscope (SEM) image of the fabricated AML, scale bar: 200 nm. (**b**) Optical image of the AML. Scale bar: 25 μm. (**c**) Measured intensity profiles of the reflected beam by the AML in the xz-plane at different wavelengths. (**d**) Simulated intensity profiles of the reflected beam by the AML in the xz-plane at different wavelengths. (Reproduced from [32] with permission.)

With the significant progress in nano-fabrication, researchers not only focus on high-resolution imaging but also exploit the diffractive optics system by overcoming more fundamental problems, such as chromatic aberration. Like the traditional refractive lenses, new techniques have been demonstrated for focusing on multiple different wavelengths’ incident light with the same focal length. Traditionally, for apochromatic and super-achromatic lenses [30], macro- or meso-scale manufacturing methods are complex. However, the metasurface provides a new approach because the flexible shape of the electromagnetic field [31] can be modified by changing the phase profile. Capasso’s group [32] reported that the phase realization process and interference mechanism result in large chromatic aberrations in diffractive lenses. For the dielectric-based metalens [5], the phase shifter, nanopillars, acts as a truncated waveguide with predetermined dispersion. To realize achromatic metalenses, the key is to optimize the phase shifters’ geometric parameters (Figure 5a) by satisfying the phase coverage from 0 to 2π with different wavelength dispersion [33]. The fabricated achromatic metalens (AML), shown in Figure 5b, has more advantages and overcomes the existing optics problems. The primary goal is to maximize the phase coverage. Second, the phase shifters guarantee polarization-insensitive operation for AML. To characterize the performance of the AML, the focal distance for different wavelength incident light was measured (Figure 5c). The simulated focal point intensity is shown in Figure 5d. Even for different wavelength incident light, the focal spots have a perfect shape at the same distance (*z* = 485 µm). The results show a theoretical and experimental achromatic response, where the focal length remains unchanged with a broad bandwidth (60 nm) in the visible range.

**Figure 6:**
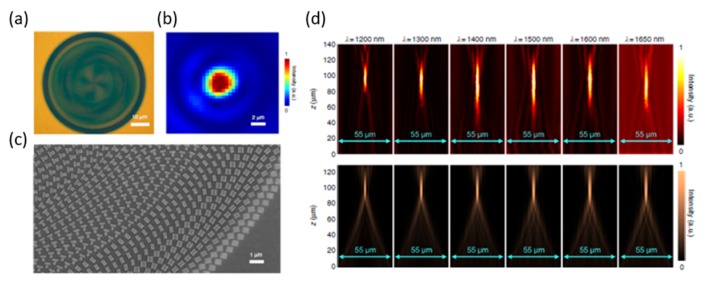
Verification of achromatic converging metalens. (**a**) Optical image of a fabricated metalens with NA = 0.268. (**b**) Measured light intensity of focal spot at incident wavelength λ = 1500 nm. (**c**) Zoomed-in scanning electron microscope (SEM) image of the fabricated metalens. (**d**) Experimental (top row) and numerical (bottom row) intensity profiles of BAML along axial planes at various incident wavelengths. (Reproduced from [35] with permission).

When a longer wavelength incident light is used in the miniaturized optical system, it will cause chromatic dispersion [34] because the index of refraction decreases with a longer wavelength. The refractive lenses need to have a larger focal length and prisms, which will deflect at a smaller angle for a longer wavelength. A serious chromatic aberration will degrade the system performance, especially in multi-color imaging applications. Although there have been some good achromatic metalens designs, which can suppress the chromatic effect over 60 nm bandwidth in the visible range [32], the working achromatic bandwidth (~11.4% of the central wavelength) is still not broad enough for practical applications. Wang et al. [35] demonstrated a new broadband achromatic metalens (BAML) (Figure 6) in the infrared (IR) range. The new metalens works within a broad infrared bandwidth at wavelengths from 1200 nm to 1680 nm.

Generally speaking, the dispersion can be divided into two different effects: dispersion elimination and dispersion expansion. For imaging purposes, researchers attempt to eliminate the dispersion because it could cause chromatic aberration and degrade the overall image quality. However, the dispersion can be used to suppress the nonlinear effects during fiber communication [36]. For natural materials, dispersion is determined by their own electronic and energy levels [37]. To resolve this problem, Li et al. [38] from the Chinese Academy of Sciences proposed a new method to control the dispersion. The metalens design was fabricated by silicon nanocuboids, which respond to specific wavelengths (473, 532, and 632.6 nm). With the Pancharatnam‒Berry (P‒B) phase shift design, the chromatic dispersion among different wavelengths can be engineered independently. Subsequently, a series of flat optical devices with both achromatic and super-dispersive (positive or negative) focusing properties can be demonstrated.

**Figure 7:**
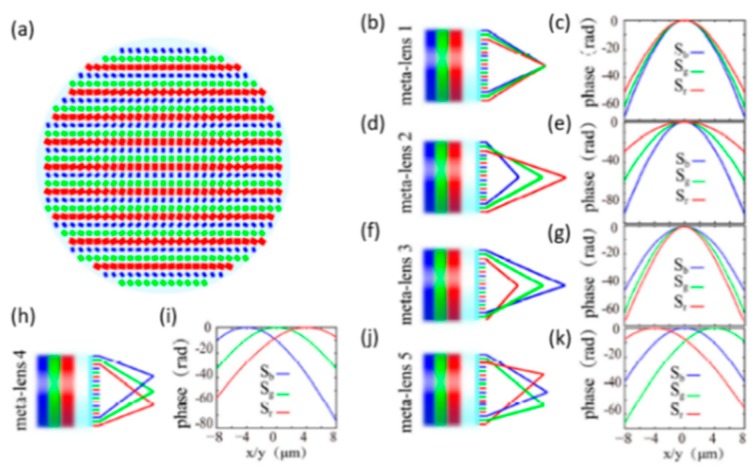
Dispersion controlling meta-lens. (**a**) The schematic of the designed metasurface. (**b**) Schematic and (**c**) phase distribution of achromatic metalens M1. (**d**) Schematic and (**e**) phase distribution of super-dispersion metalens M2 designed to separate different wavelength light in order of normal dispersion. (**f**) Schematic and (**g**) phase distribution of super-dispersion flat metalens M3 with anomalous dispersion. The distribution of focal points is opposite to M3. (**h**) Schematic and (**i**) phase distribution of super-dispersion flat metalens M4 with off-axis color separation. (**j**) Schematic and (**k**) phase distribution of super-dispersion flat metalens M5 with different off-axis colors separation. (Reproduced from [38] with permission).

As shown in Figure 7a, there are five designed metasurface lenses. The first metalens M_1_ is a flat achromatic lens (*f* = 10 μm and NA = 0.6294). The schematic and phase profile of M_1_ are shown in Figure 7b,c. The second and third metalenses (M_2_ and M_3_) were designed as super-dispersion metalenses, shown in Figure 7d–g. For M_2_, it could focus the red, green, and blue light into different focal lengths (6, 10, and 17 μm). M_3_ has a reversed super-dispersion where focal points of red, green, and blue light are in reverse order compared to M_2_. M_4_ and M_5_ are off-axis super-dispersion metalenses, shown in Figure 7h–k).

In Table 2, it shows the different achromatic metasurface-based lens designs. The broadband achromatic lenses are pursued by researchers, with the great development of nano-fabrication technique and new materials applied in optical system, the performance of designed metasurface-based lenses will have a remarkable breakthrough.

### 3.3. Metalens-Enabled Focus Scanning Devices

**Figure 8:**
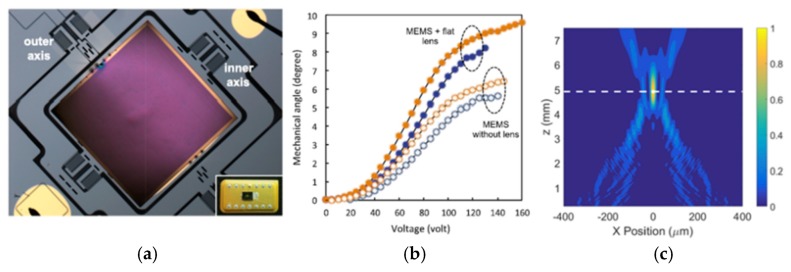
Metasurface-enabled MEMS (**a**) Optical microscope image of a MEMS scanner with a flat lens on top. (**b**) Angular displacement of the MEMS scanner with and without the metasurface-based lens. (**c**) Simulation: distribution of the intensity of the reflected beam in the *xz*-plane at *y* = 0. The maximum intensity occurred when *z* = 5 mm and focusing efficiency is 83%. (Reproduced from [47] with permission.)

Miniaturized optical imaging and sensing systems commonly require focusing scanning or tuning mechanisms with ultra-compact size, lightweight, and fast scan speed. Micro-electro-mechanical system (MEMS) [39,40], electrowetting [41,42] and thermal tuning-based [43] methods have provided partial solutions. However, so far, these approaches are still relatively bulky and only provide a relatively slow tuning speed. Tunable focusing liquid crystal lenses [44,45,46] have been proposed for high tuning speed. However, due to the polarization dependence, the tuning speed is fully restricted. Metalenses have shown great potential for focus tuning applications. A reflective-mode metalens has been successfully integrated onto a high-speed MEMS scanner through the collaboration between Capasso’s group at Harvard University and Lopez’s group at the Argonne National Lab [47] for lateral beam scanning. Actuated by staggered comb-drives, the 2D MEMS scanner offers fast speed beam steering, high-resolution imaging, and high optical efficiency [48]. The fabricated metasurface lens was on a square substrate with side 0.8 mm and has a focal length of 5 mm with incident light 45° away from the surface, shown in Figure 8a. The metasurface lens was mounted at the center of the 2D MEMS scanner. To achieve biaxial scanning, the outer gimbal-frame rotates at a slow speed while the inner mirror scans with 9° tilting angle at high speed (~1 kHz). With an increasing voltage applied on the rotational axis, the MEMS scanner begins rotating until it reaches the maximum angles. The relationship between the applied voltage and the mechanical angle is shown in Figure 8b. To characterize the performance of the metasurface lens, the Finite Difference Time Domain (FDTD) method has been used to search for the electric field distribution (Figure 8c). 

**Figure 9:**
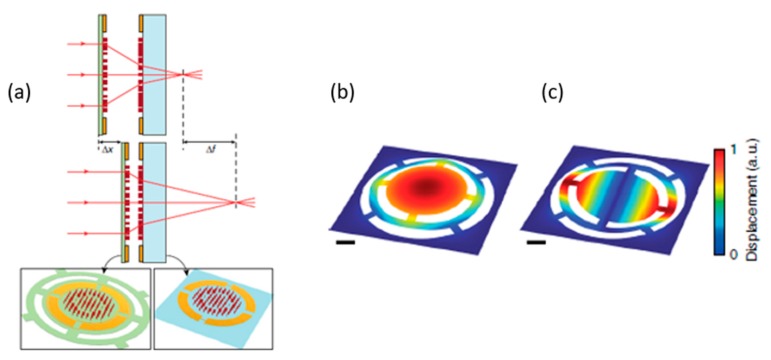
Metasurface-based MEMS device wit tunable focus. (**a**) Schematic illustration of the proposed tunable lens, comprised of a stationary lens on a substrate, and a moving lens on a membrane. The first (**b**) and second (**c**) mechanical resonances of the membrane at frequencies of 2.6 and 5.6 kHz, respectively. The scale bars are 100 µm. (Reproduced from [49] with permission.)

Another metasurface-based MEMS device with tunable focus has been reported by Faraon’s group [49] at Caltech. The new MEMS device was composed of two metalenses (a converging and diverging metasurface lenses), shown in Figure 9a. An axial focus scanning length can be achieved within a range of 565 to 629 µm. The first stationary metasurface lens was fabricated on a glass substrate and another moveable metasurface was fabricated on the SiN*_x_* membrane. By applying a voltage potential, the distance between the two metalenses will change. By calculation, the various distance (Δx ~1 µm) results in a large tuning range (Δf ~ 36 µm). To tunable focal length, two series capacitors were placed on the substrates. In Figure 9b,c, the mechanical resonance of 2.6 kHz and 5.6 kHz was measured. 

**Figure 10:**
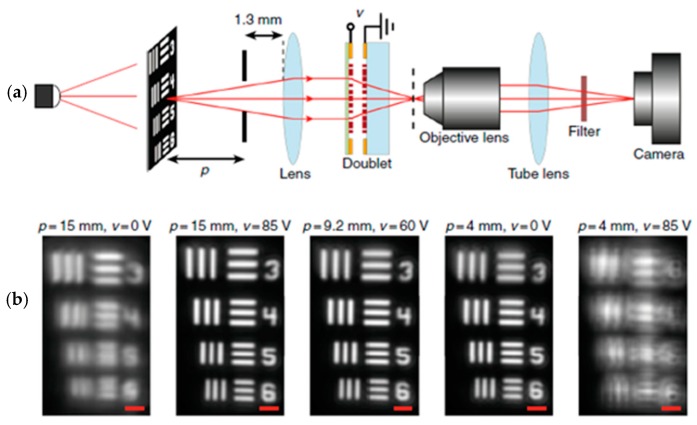
Imaging with the tunable doublet. (**a**) Schematic illustration of the imaging setup using a regular glass lens and the tunable doublet. The image formed by the doublet is magnified and re-imaged using a custom-built microscope with a ×55 magnification onto an image sensor. (**b**) Imaging results, showing the tuning of the imaging distance of the doublet and glass lens combination with applied voltage. By applying 85 V across the device, the imaging distance increases from 4 to 15 mm. The scale bars are 10 µm. (Reproduced from [49] with permission.)

For testing the image qualities, the measurement step-up is shown in Figure 10a. When the object sets p ~ 15 mm away from the lens and no voltage applied, the image was out of focus. When we increased the voltage to 85 V, the image became clear. The same measurement was done for p equals 4 and 9.2 mm (Figure 10b).

### 3.4. Computational Optics Based on Metasurface

For bio-imaging applications, the most challenging problem is to image through scattering media (like human tissue specimens), because the passing light may have a complex speckle pattern. There are many existing methods to achieve high-resolution imaging, such as wavefront engineering [50], speckle correlations based on speckle correlations via non-invasive imaging through scattering layers [51,52], and a transmission matrix [53]. Recently, multiple computational imaging approaches have been demonstrated, including phase-space measurements [54,55], wavefront sensing with the Demon algorithm [56], and the speckle-correlation scattering matrix (SSM) [57,58]. For the conventional scattering media (CSM), lots of limitations still exist for practical applications in the real world, such as the stability of optical properties [59] and incomplete channel control in multiple scattering [60]. The trade-off between maximum scattering angle and memory-effect range [27] will ultimately cause significant defects from a practical point of view.

**Figure 11:**
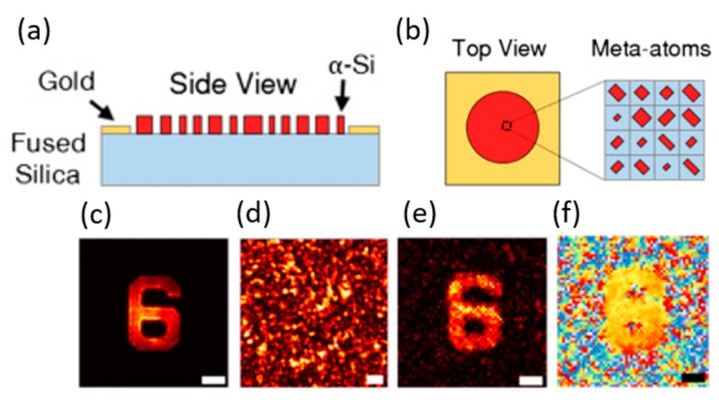
Metasurface-based computational imaging process. (**a**) Schematic illustration of the side view of the MD. (**b**) Schematics of a uniform array and unit cell of the metasurface, showing the parameter definitions. The transmission phase of the two orthogonal polarizations can be manipulated using the meta-atoms. (**c**) In-focus images of targets captured by a custom-built microscope. (**d**) The resulting speckle patterns of the samples after passing through the MD. (**e**) The retrieved object amplitudes. (**f**) Phases from the captured speckle patterns. The scale bars are 25 µm. (Reproduced from [64] with permission.)

Recently, a few studies have been reported on the metasurface diffuser (MD), which can be used in wavefront control with a spatial light modulator [2,5,61,62,63]. The results show that the new imaging system will obtain a large FOV and high-resolution imaging quality. However, speckle patterns’ computational imaging is less studied. Faraon’s group [64] proposed a method combining MDs and SSM to replace the CSM complex field for 3D imaging. Researchers indicated that the MDs (Figure 11a) capture samples’ amplitude and holographic imaging with numerical backpropagation. The nano-scatterers array (Figure 11b) provides 2π phase coverage and NA = 0.6.

To characterize the metasurface-based computational imaging system, the 1951 USAF resolution test target was used as an amplitude object (Figure 11c–f). As a result, CSM can be replaced by SSM and MD will overcome the trade-off limitations between efficiency and scattering angle.

## 4. A Metalens -Based Optical Imaging and Sensing System

Metalenses have been successfully integrated into state-of-the-art miniaturized bio-optical systems. Experimental results have validated the feasibility of the metalens for very broad applications in the future. The metalens holds promise for miniature optical imaging and sensing systems, such as optical coherence tomography (OCT), two-photon fluorescence microscopy, confocal microscopy, and spectrometers (Table 3). Details will be introduced in each session.

### 4.1. Metalens-Based Endoscopic Optical Coherence Tomography (OCT)

Endoscopic optical coherence tomography (OCT) [65,66] has been developed as a non-invasive bio-imaging tool for early cancer detection, diagnosis, and cancer staging. Among the various bio-imaging modalities, confocal endomicroscopy can provide high resolution of tissue structures at the cellular level but lacks sufficient penetration depth. In contrast, OCT provides an adequate depth range (up to 1 mm) and FOV but the limited resolution (relatively lower lateral resolution). OCT was designed based on coherence interferometry [67,68], which obtains images from under-the-surface tissue structures [69,70]. New OCT tools with ultra-high image resolution have been demonstrated [71,72,73,74,75,76,77]. Shorter wavelengths with broader-bandwidth light sources potentially improve image resolution. However, the depth of penetration still does not meet the requirements for applications such as deep tissue scanning. Capasso’s group and Suter’s group at Harvard University have recently developed a new metalens-based endoscopic OCT [78] that can provide high-resolution imaging with extended depth. 

**Figure 12:**
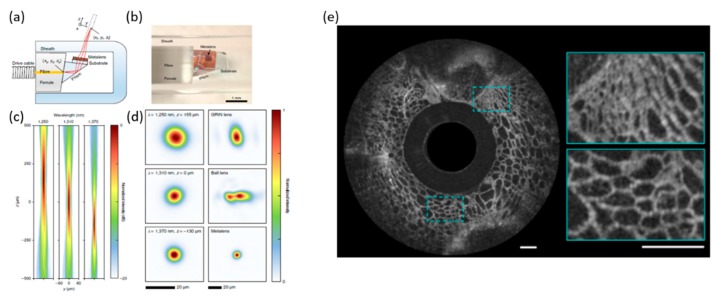
Metalens-based endoscopic optical coherence tomography (**a**) Schematic of the nano-optic endoscope. (**b**) Photographic image of the distal end of nano-optic endoscope. (**c**) Measured intensity distribution of the output beam of the nano-optic endoscope along the propagation direction in the yz-plane at λ = 1250, 1310 and 1370 nm. (**d**) (**left**) Focal spot profiles of the nano-optic endoscope at corresponding wavelengths. (**d**) (**right**) Focal spot profiles of a graded-index (GRIN) OCT catheter, a ball lens OCT catheter and the nano-optic endoscope at 1310 nm wavelength. (**e**) OCT images of fruit flesh (grape) obtained using a nano-optical endoscope. (Reproduced from [78] with permission.)

As shown in Figure 12, the new OCT endoscope uses the metalens to replace the refractive lenses. However, the chromatic dispersion is a large defect in the system. Several techniques [79,80] have been reported to overcome or reduce the chromatic dispersion of metasurface. A phantom with a subwavelength gold line on the glass substrate was fabricated by electron-beam lithography (EBL). This new nano-optic endoscope was connected to the Fourier–domain OCT system [81,82]. The effective depth of focus will achieve 211 µm (tangential) and 315 µm (sagittal), larger than the achromatic lens, with the same NA that can provide (about 90 µm). Ex vivo imaging on freshly excised lung tissue has been demonstrated with the metalens-based nano-optic endoscope (Figure 12e), visualizing fine features in tissue, including epithelium, basement membrane, and cartilage.

### 4.2. Metalens-Based Two-Photon Microscope

**Figure 13:**
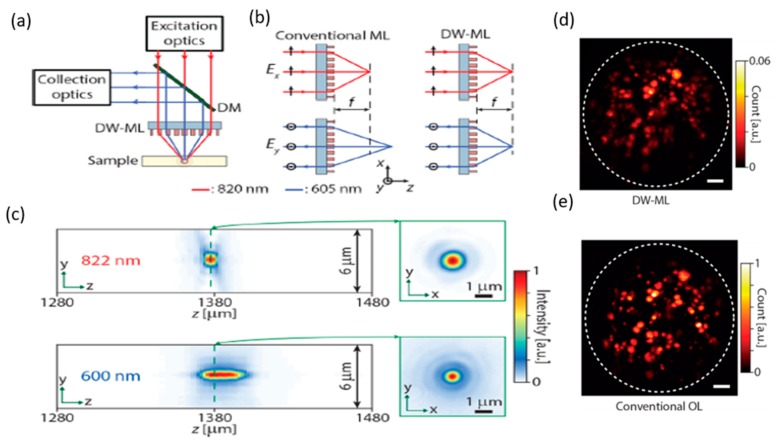
Metalens-based two-photon microscope system. (**a**) Schematic of a two-photon microscope employing a metasurface objective. (**b**) Schematic illustration of a conventional metasurface lens focusing light with different wavelengths to distinct focal lengths, and the DW-ML designed to focus 820 nm *x*-polarized light and 605 nm *y*-polarized light to the same focal distance of f. (**c**) (**Top**) Measured light intensity in the axial (**left**) and focal (**right**) planes for *x*-polarized 822 nm illumination. (**Bottom**) Same results as in the top figure for a *y*-polarized light source with a center wavelength of 600 nm and full width at half-maximum of approximately 10 nm. (**d**) Two-photon fluorescent microscope image captured by DW-ML. (**e**) captured using a conventional refractive objective, scale bars: 10 µm. (Reproduced from [91] with permission).

In biological study, high-resolution two-photon microscopy (nonlinear optics) has been widely used [83,84,85,86]. The development of a miniaturized two-photon microscope is still a challenge in the optics field. With the progress in dielectric metasurface research, researchers in Caltech have begun to construct a new two-photon microscope with tiny metalenses, because of the unique advantages such as lightweight, small structure, high efficiency, and controllable phase profiles. Commonly, metalenses are used for single-photon (linear optics) fluorescence images due to their limits with narrow effective wavelength. Metalenses usually operate at a single wavelength. However, for fluorescence microscopic imaging, metalenses will have excitation and emission wavelengths at different focal positions due to the chromatic dispersion [87,88,89,90]. This dispersion will reduce the collection efficiency of the system. A metalens-based two-photon microscope has been proposed by Arbabi et al. [91]. The designed system used double-wavelength metalenses (DW-ML) to replace the objective lenses. The DW-ML has the excitation and emission wavelength at the same focal distance and can acquire high-resolution fluorescence images compared to the traditional objectives. A schematic drawing of the metalens-based two-photon microscope is shown in Figure 13a. The incident high-peak-power pulsed laser passes through the DW-ML and is focused into the specimens. Collimated by the DW-ML, emitted fluorescent light (shorter wavelength) will be reflected by the dichroic mirror and collected by a detector. To realize the same focal length, the birefringent dichromic meta-atom method has been used [92]. The excitation wavelength is 820 nm near-infrared (NIR), while the collected emission light wavelength is 605 nm (Figure 13b). The nanofins are based on polycrystalline silicon (p-Si) due to its high index and low efficiency loss at the selected wavelength. Figure 13c shows the imaging characterization. Finally, the new metalens has been tested by replacing the traditional objectives in a table-top two-photon microscope. The acquired images of fluorophore-coated polyethylene microspheres are shown in Figure 13d,e. The quality of the DW-ML image is similar to that of conventional and bulky objectives. Although the DW-ML can provide a high-resolution image for a two-photon microscope, the number of collected photons is 15× lower and the required excited laser energy is 4.7× higher than a conventional objective.

### 4.3. Metalens-Based Confocal Microscope System

**Figure 14:**
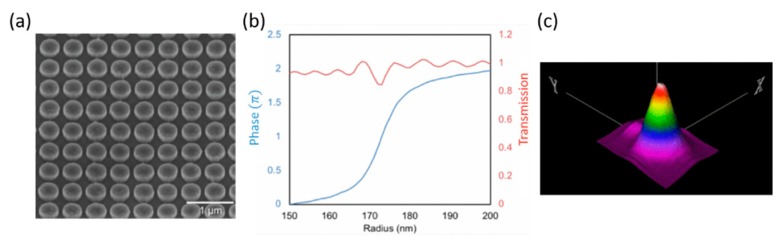
Metalens-based confocal microscope system. (**a**) TiO_2_ nanoresonator array. (**b**) Phase and transmission vs. nanodisc radius by FDTD simulation. (**c**) a 3D intensity laser-focused spot profile. (Reproduced from [93] with permission.)

The metalens has become an emerging technology for advanced miniature bio-medical optical imaging systems, including confocal microendoscopy for imaging hollow organs, which is one of the most promising imaging tools used in the clinic for “optical biopsy” applications. Qiu et al. [93] of Michigan State University recently demonstrated the feasibility of using a metalens for miniaturizing an optical fiber-based laser confocal microscope. The Huygens’ metasurface-based metalens [94,95] has been made out of TiO_2_ nano-disc shape resonators, which are polarization-independent (Figure 14a). With the support of crossed electric and magnetic dipole resonances, the incidence scattering direction will be cancelled, leaving only the forward propagation. By the ideal of designing the nanostructure geometry, the resonator arrays are able to achieve high transmission efficiency and 2π phase shift coverage (Figure 14b). The confocal microscope system consists of a photodetector, a data acquisition system, a CW incident laser wavelength of 660 nm, and Huygens’ metalens. The system was characterized using the USAF resolution target. The measured focal length is 4 mm and the focused spot size is 4.29 µm (Figure 14c).

### 4.4. Metalens-Enabled Advanced Spectrometer

A miniaturized spectrometer system is another important and promising application for metalenses. Advanced spectrometers have been utilized for many applications, such as on-site environmental monitoring and in vivo disease diagnostics [96,97,98]. Currently, spectrometers are developed with conventional optics, such as non-polarizing beam splitters, wave plates, and polarizers. These spectrometer systems usually consist of focusing mirrors and grating turret [99]. Because of the limited grating dispersion, large spatial separation is hard to achieve within a short light propagation distance. Due to the limited properties of traditional optics, researchers are motivated to search for novel optics, such as metasurface-based lenses.

**Figure 15:**
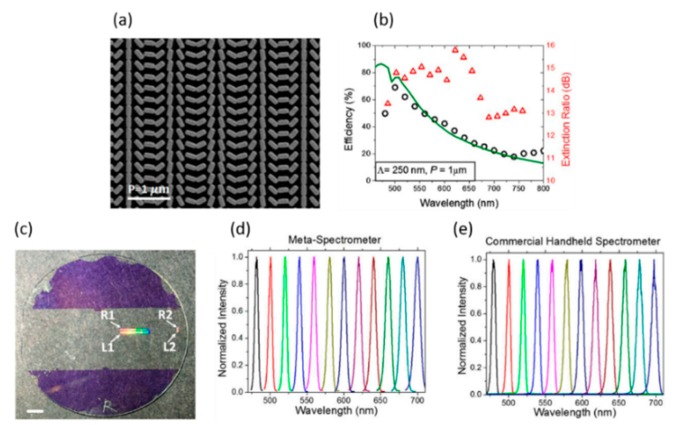
Ultra-compact visible chiral spectrometer with metalens. (**a**) Scanning electron microscope image of a fabricated off-axis metalens. (**b**) Measured (**black circles**) and simulated (**green line**) efficiencies of the +1 order metasurface grating under illumination with right-handed circularly polarized light. Extinction ratio (**red triangles**) of the meta-grating as a function of wavelength. (**c**) Photograph of a fabricated device with four separate metalenses labeled R1, R2, L1, and L2. (**d**) Measured spectra from a supercontinuum laser with 5 nm bandwidth using the metalens spectrometer. (**e**) Measured spectra from a commercial handheld spectrometer, the center wavelength varied from 480 nm to 700 nm. (Reproduced from [101] with permission.)

A new silicon-based off-axis metalens at near-infrared (NIR) wavelength has been demonstrated, which can provide higher spectral resolution than the conventional grating [100]. To improve the efficiency in the visible range, Capasso’s group [101] also proposed an upgraded system, which operates in the visible wavelength range (Figure 15), adding an additional process—titanium oxide atomic layer deposition (ALD) [102]. The meta-spectrometer has several advantages: (1) focusing and dispersive elements will be placed in a planar structure; (2) it can surpass traditional blazed grating such as larger dispersions; (3) with different NA metasurface structures integrated on a single substrate, multiple spectral resolutions can be achieved.

**Figure 16:**
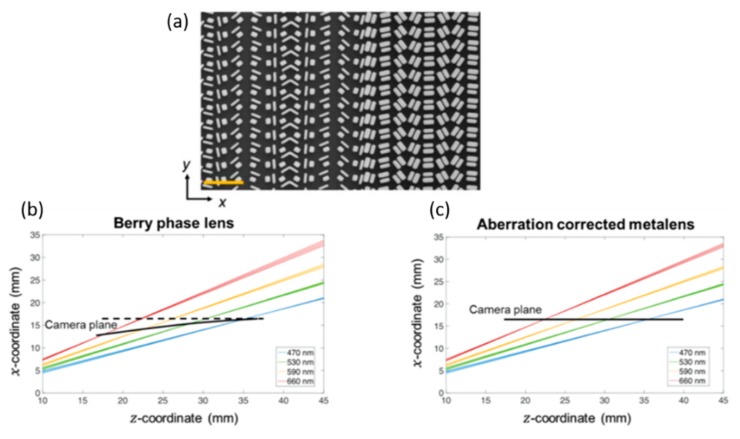
Compact aberration-corrected spectrometer. (**a**) Scanning electron micrographs of a fabricated aberration-corrected off-axis metalens. (**b**) A regular Berry-phase lens and (**c**) an aberration-corrected metalens. The metalenses designed with focal length f = 40 mm and focusing angle a = 25 degrees at wavelength λ = 470 nm. The focusing planes for each case are indicated by bold lines; the dashed line in the left is horizontal and meant as a reference to the curved focal plane. (Reproduced from [107] with permission.)

The challenges of refractive and diffractive limitations [103,104,105,106] still exist, such as curved focal plane and different wavelength aberrations, for the off-axis focusing metalenses. To resolve these, a newly developed aberration-corrected off-axis metalens [107], shown in Figure 16a, has a focal spot profile with a broad bandwidth, simultaneously engineering the phase and its higher order derivatives with respect to frequency (i.e., group delay (GD) and GD dispersion (GDD)). To achieve off-axis focusing, the phase profile of the metalens needed to satisfy Equation (3):

(3) $$\varphi \left(x,y,\omega \right)=-\frac{\omega }{c}\left(\sqrt{{\left(x-x_{f}\right)}^{2}+y^{2}+{\left(z-z_{f}\right)}^{2}}-f\left(\omega \right)\right)$$

where *x* and *y* are spatial coordinates along the lens, and ω is the angular frequency of incident light. c is the light speed in vacuum. By using Taylor expanding on Equation (2) at designed angular frequency $\omega_{d}$, the phase profile can be represented by Equation (4):

(4) $$\varphi \left(x,y,\omega \right)=\varphi \left(x,y,\omega_{d}\right)+\frac{\partial \varphi }{\partial \omega }\cdot \left(\omega -\omega_{d}\right)+\frac{\partial^{2}\varphi }{2\partial \omega^{2}}\cdot {\left(\omega -\omega_{d}\right)}^{2})$$

where the second term $\frac{\partial \varphi }{\partial w}$ of the partial derivative is the GD and the next higher term $\frac{\partial^{2}\varphi }{\partial \omega^{2}}$ represents GDD. The working distance is 4 cm and the spectral resolution can achieve 200 nm bandwidth in the visible range. To characterize the metalens performance, a traditional Berry phase lens was used as the comparison target. Each lens was illuminated with a collimated monochromatic laser at different wavelengths: 488, 532, 632, and 660 nm (Figure 16b).

**Figure 17:**
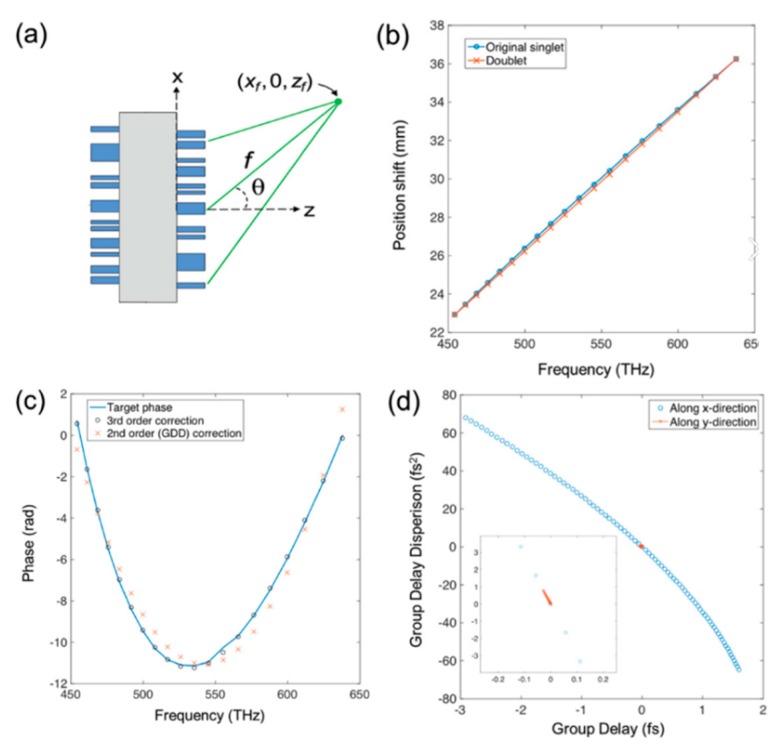
Compact aberration-corrected spectrometer characterizations. (**a**) Schematic of a doublet, comprising of a metasurface corrector and the original aberration-corrected off-axis metalens. The corrector serves to impart group delay (GD) and group delay dispersions (GDDs) such that the focal spot positions of the metalens are linear in frequency. (**b**) Plots of the focal spot positions of the singlet metalens (**blue**) and the doublet (**orange**) as a function of frequency. (**c**) Plot of the required phase as a function of frequency for an element at the edge of the metasurface corrector (**blue line**), and the results of second- and third-order polynomial fits (**orange crosses** and **black circles**, respectively). (**d**) GD and GDD values required for elements across the middle of the metasurface corrector, along *x* and *y* directions (**blue circles** and **orange crosses**). Inset: magnified view of the dispersion required for elements along *y*, which is minimal, due to the choice of the orthogonal camera plane. (Reproduced from [107] with permission.)

The focal spots of the aberration-corrected metalens have the perfect single-peak signal for four different wavelengths. However, the focal spots for Berry phase lens become abnormal when the wavelength exceeds 532 nm. Another challenge for the aberration-corrected metalens is the dispersion. A doublet-based metalens has been micro-machined to achieve strictly linear dispersion (Figure 17). The spectrometer can work for the helicity-polarized light.

**Figure 18:**
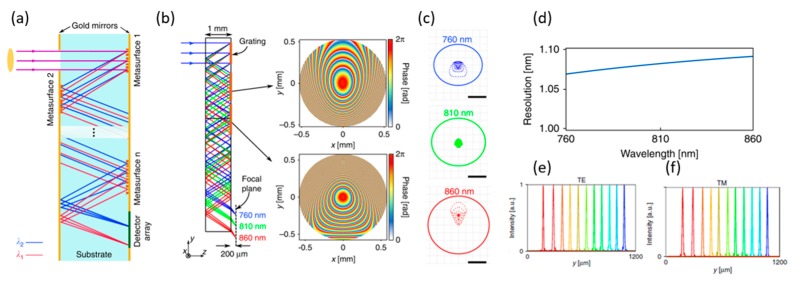
Compact folded metasurface spectrometer (**a**) The proposed scheme for a folded compact spectrometer. (**b**) Ray-tracing simulation results of the folded spectrometer, shown at three wavelengths in the center and two ends of the band. The system consists of a blazed grating that disperses light to different angles, followed by two metasurfaces optimized to focus light for various angles (corresponding to different input wavelengths). The grating has a period of 1 μm, and the optimized phase profiles for the two metasurfaces are shown on the right. (**c**) Simulated spot diagrams for three wavelengths: center and the two ends of the band. The scale bars are 5 μm. (**d**) Spectral resolution of the spectrometer, which is calculated from simulated Airy disk radii and the lateral displacement of the focus with wavelength. (**e**,**f**) One-dimensional focal spot profiles measured for several wavelengths in the bandwidth along the y-direction (as indicated in the inset) for TE and TM polarizations. The wavelengths start at 760 nm (blue curve) and increase at 10-nm steps up to 860 nm (red curve). (Reproduced from [106] with permission.)

The compact spectrometer design successfully integrates a series of different wavelength band-pass filters on a single array of photodetectors [108,109]. However, such devices have system limitations in terms of resolution due to filter quality factors. Another design based on flat on-chip photonics can provide high spectral resolution [110,111,112,113,114,115], but the input on-chip coupling light losses and the reduced output are major challenges in many applications. To achieve a compact spectrometer with high resolution, Faraon’s group [106] proposed a compact folded multi-metalenses system. With only 1 mm thickness, the new spectrometer can provide 1.2 nm resolution over a broad bandwidth range (~ 100 nm) in the NIR range. A schematic is shown in Figure 18a. Multiple metalenses are micro-machined on a transparent substrate to focus and disperse the incident light to different points. Ray-tracing simulation results are shown in Figure 18b, while the spot profiles are shown in Figure 18c. A small aberration was confirmed in the range of 760 nm to 860 nm. Using the diffraction-limited Airy radius and the focus displacement by the wavelength, the resolution was calculated in Figure 18d; the theoretical value is ~1.1 nm. To characterize the performance of the designed system, one-dimensional intensity profiles are shown in Figure 18e,f for transverse electric (TE) and transverse magnetic (TM).

## 5. Conclusions and Future Work

Metalenses has shown great potential for the development of miniaturized imaging and sensing systems. By replacing traditional lenses of bulky size, researchers have successfully demonstrated not only novel metalens designs but also advanced metalens-based optical systems with ultra-compact size. Many bio-optical applications will benefit from the remarkable advantages of the metalens, including ultrathin structure, large FOV, achromatic effects in a broad bandwidth, etc. Superior optical performance can be achieved by optimizing nanostructures on the metalens’ surface. It has been proven that a metalens could potentially have a very high transmission or reflectivity efficiency. We believe that metalenses will have significant impacts on multiple imaging and sensing modalities, such as camera-based imaging systems, optical coherent tomography, two-photon, confocal, and spectrometer.

In a miniaturized optical system, metalenses could provide plenty of functions, including phase control, polarization, focus tuning, etc. Currently, most studies are still mainly focused on the fundamental properties of metalenses and their advanced microfabrication. In the future, metalens-based miniaturized bio-optical systems will attract more attention for broader biomedical applications, such as handheld, wearable, endoscopic, and implantable medical devices for quantitative healthcare.

## Figures and Tables

**Table 1:** Comparison between each fabricated metasurface-based lens in wide angular FOV design.

Reference (Year)	Efficiency	Material	NA	Wavelength	FOV
Arbabi et al. (2016) [24]	70%	a-Si:H	N/A	850 nm	±30°
Groever et al. (2017) [25]	N/A	TiO_2_	0.44	532 nm	±25°
Jang et al. (2018) [27]	N/A	SiN*_x_*	>0.5	532 nm	8 mm
Guo et al. (2018) [28]	93%	Simulation	0.89	Far-field power	±60°
Xu et al. (2018) [29]	95%	SiN*_x_*	N/A	532 nm	±80°

**Table 2:** Comparison of metasurface-based lens in broadband achromatic optics design.

Reference (Year)	Material	NA	Wavelength
Khorasaninejad et al. (2017) [32]	TiO_2_	0.2	490–550 nm
Wang et al. (2017) [35]	Au	0.324	1200–1680 nm
Li et al. (2017) [38]	Si	0.629	473, 532, and 632.8 nm

**Table 3:** Metalens-based optical imaging and sensing system.

Reference (Year)	Modality	Wavelength
Pahlevaninezhad et al. (2018) [78]	OCT	λ_Ex_ = 1310 nm
Arbabi et al. (2018) [91]	Two-photon Fluorescence	λ_Ex_ = 820 nm
Qiu et al. (2018) [93]	Confocal	λ_Ex_ = 660 nm
Zhu et al. (2017) [101]	Spectrometer	480–700 nm
